# Insecticidal Effect of Entomopathogenic Nematodes and the Cell-Free Supernatant from Their Symbiotic Bacteria against *Philaenus spumarius* (Hemiptera: Aphrophoridae) Nymphs

**DOI:** 10.3390/insects12050448

**Published:** 2021-05-14

**Authors:** Ignacio Vicente-Díez, Rubén Blanco-Pérez, María del Mar González-Trujillo, Alicia Pou, Raquel Campos-Herrera

**Affiliations:** Instituto de Ciencias de la Vid y del Vino (CSIC, Gobierno de La Rioja, Universidad de La Rioja), 26007 Logroño, Spain; ignacio.vicente@icvv.es (I.V.-D.); ruben.blanco@icvv.es (R.B.-P.); mariadelmar.gonzalez@icvv.es (M.d.M.G.-T.); apou@larioja.org (A.P.)

**Keywords:** biocontrol, *Heterorhabditis*, *Photorhabdus*, *Steinernema*, sustainable agriculture, *Xenorhabdus*, *Xylella fastidiosa*

## Abstract

**Simple Summary:**

The disease caused by *Xylella fastidiosa* affects economically relevant crops such as olives, almonds, and grapevine. Since curative means are not available, its current management principally consists of broad-spectrum pesticide applications to control vectors like the meadow spittlebug *Philaenus spumarius*, the most important one in Europe. Exploring environmentally sound alternatives is a primary challenge for sustainable agriculture. Entomopathogenic nematodes (EPNs) are well-known biocontrol agents of soil-dwelling arthropods. Recent technological advances for field applications, including improvements in obtaining cell-free supernatants from EPN symbiotic bacteria, allow their successful implementation against aerial pests. Here, we investigated the impact of four EPN species and their cell-free supernatants on nymphs of the meadow spittlebug. First, we observed that the exposure to the foam produced by this insect does not affect the nematode virulence. Indeed, direct applications of certain EPN species reached up to 90–78% nymphal mortality rates after five days of exposure, while specific cell-free supernatants produced 64% mortality rates. Overall, we demonstrated the great potential of EPN and cell-free supernatant of their symbiont bacteria applications against this vector, opening new venues to develop novel biopesticides for integrated management practices and organic productions.

**Abstract:**

The meadow spittlebug *Philaenus spumarius* (Hemiptera: Aphrophoridae) is the primary vector of *Xylella fastidiosa* (Proteobacteria: Xanthomonadaceae) in Europe, a pest–disease complex of economically relevant crops such as olives, almonds, and grapevine, managed mainly through the use of broad-spectrum pesticides. Providing environmentally sound alternatives to reduce the reliance on chemical control is a primary challenge in the control of *P. spumarius* and, hence, in the protection of crops against the expansion of its associated bacterial pathogen. Entomopathogenic nematodes (EPNs) are well-known biocontrol agents of soil-dwelling arthropods. Recent technological advances in field applications, including improvements in obtaining cell-free supernatant from their symbiotic bacteria, allow their successful implementation against aerial pests. Thus, this study aimed to evaluate, for the first time, the efficacy of EPN applications against nymphal instars of *P. spumarius.* We tested four EPN species and the cell-free supernatant of their corresponding symbiotic bacteria: *Steinernema feltiae*–*Xenorhabdus bovienii*, *S. carpocapsae*–*X. nematophila*, *S. riojaense*–*X. kozodoii*, and *Heterorhabditis bacteriophora*–*Photorhabdus laumondii* subsp. *laumondii*. First, we showed that 24 and 72 h exposure to the foam produced by *P. spumarius* nymphs did not affect *S. feltiae* virulence. The direct application of steinernematid EPNs provided promising results, reaching 90, 78, and 53% nymphal mortality rates after five days of exposure for *S. carpocapsae*, *S. feltiae*, and *S. riojaense*, respectively. Conversely, the application of the cell-free supernatant from *P. laumondii* resulted in nymphal mortalities of 64%, significantly higher than observed for *Xenorhabdus* species after five days of exposure. Overall, we demonstrated the great potential of the application of specific EPNs and cell-free supernatant of their symbiont bacteria against *P. spumarius nymphs*, introducing new opportunities to develop them as biopesticides for integrated management practices or organic vineyard production.

## 1. Introduction

The xylem-inhabiting Gram-negative bacterium *Xylella fastidiosa* (Proteobacteria: Xanthomonadaceae) can damage several relevant crops that affect the global farming economy. The main problem associated with these diseases is the obstruction of the xylem, with symptoms ranging from leaf marginal necrosis and leaf abscission to dieback, delayed growth, and death of plants through insufficient water flow [1,2]. The current forecast for the expansion and severity of these diseases, named as the grapevine Pierce’s disease (PD) or the Olive Quick Decline Syndrome (OQDS), may increase shortly [1,2,3], but they are characterized by symptoms often similar to water stress [4]. The bacterium *X. fastidiosa* is known to colonize crops of different climatic zones worldwide. Its presence has already been reported in several countries in the EU, including Italy (west coast of Salento Peninsula, Apulia, and the Argentario, Tuscany), France (the island of Corsica and the Provence-Alpes-Côte d’Azur region), Portugal (district of Porto), and Spain (Madrid, Alicante, and the Balearic Islands) [5].

The meadow spittlebug *Philaenus spumarius* (Hemiptera: Aphrophoridae) is considered the principal vector of *X. fastidiosa* in Europe and an emergent threat for several perennial crops, including vineyards and olive and almond groves [6,7]. This xylem sap-feeding insect has a univoltine life cycle and the eggs can diapause over winter for more than one hundred days, although adults survive if the climate is appropriate [8]. The eggs usually hatch in early spring, and the five nymphal instars feed on plant shoots covered by a mucilaginous foam [9] that serves as a barrier that allows the diffusion of O_2_ from the surrounding atmosphere [10]. A recent study completed in the Iberian Peninsula has shown that this spittlebug mainly occurs in the spring season on herbaceous ground vegetation in olive groves across Southern, Eastern, and Central Spain and Northeastern Portugal [11]. However, it is likely that their populations increase and expand due to climate change [12,13]. The adults emerge after 5–8 weeks to start mating in late spring to early summer and, depending on weather conditions, oviposition begins in early November or later depending on the region [14]. The mucilaginous foam is known to protect the nymphs from desiccation and high temperatures [15] and could also fulfill other biological functions. For example, bioassays with cercopid nymphal foam revealed that it could protect them from some predators because they can be repellent or produce irritation [16]. Both the nymphal and adult instars of *P. spumarius* can inoculate the pathogen *X. fastidiosa* to healthy plants immediately after acquiring it by feeding on the xylem of infected plants [7].

Since there are currently no curative means for the control of the diseases caused by *X. fastidiosa* [4], such as PD in grapevines or OQDS in olive groves, the management of these diseases focuses on its vectors [2], mainly based on chemicals, particularly on neonicotinoids’ and pyrethroids’ products [17]. For the biological control of *P. spumarius*, there are only a few reports involving entomopathogenic fungi [18], some parasitoids, and natural predators such as wasps and spiders [19]. Under the current paradigm of severe restrictions in the use of pesticides for pest control [20,21], there is an urgent need for more biologically sound and low impact practices [22]. In this context, it is crucial to search for efficient biotools and new alternative management strategies based on biological control agents and natural compounds compatible with integrated management practices (IPM) and organic production.

Entomopathogenic nematodes (EPNs) in the families Steinernematidae and Heterorhabditidae are well-known biological control agents that become entomopathogens in symbiosis with γ-Proteobacteria species in the genera *Xenorhabdus* and *Photorhabdus*, respectively [23,24,25,26]. Their non-feeding free-living infective juvenile (IJ) stage survives in the soil, searching for a suitable host. Once located, IJs penetrate within the hemocoel to release the symbiont bacteria. The nematode–bacterium complex overcomes the host’s immune response, allowing the bacteria to proliferate exponentially and killing the arthropod by septicemia within 48–72 h after infection [23]. The IJs develop into adults and reproduce, feeding on their partner bacteria and degraded host tissues until the resources deplete. The second-stage juveniles then molt to IJs, incorporate some of the symbiont bacteria, and emerge from the host into the soil to begin a new cycle [27,28]. During this process, *Xenorhabdus* and *Photorhabdus* bacteria produce a diversity of natural products (NPs), such as phage-derived bacteriocins, colicin E3-type killer proteins, and insect toxin complexes, that kill the host and defeat other microbes competing for food sources [29,30,31]. These NPs, present in the cell-free supernatant, exhibit toxicity against many pests [32].

Traditionally, the application of EPNs was limited to the biological control of arthropod pests that inhabit agricultural soils [33,34]. Advances in application and formulation technologies allow their use against aerial pests [35]. The use of EPNs could be an alternative to manage *P. spumarius*‘s nymphs. A previous study reported that the use of native EPN species produced high nymphal mortality rates (62–73%) against the species *Philaenus simulans* and *P. teapana* in sugar cane fields [36]. However, it is unknown if *P. spumarius* control can also be effective by EPN applications. In addition, whether the foam produced by the nymphs may be a suitable environment for EPN survival is still unknown. Furthermore, even if the aerial application of cell-free supernatant is becoming a novel system to control different pests [37], it has not yet been tested against any species of spittlebug nymphs. Thereby, we hypothesized that the foam produced by *P. spumarius* nymphs might not affect EPNs, and EPN activity against *P. spumarius* nymphs will be species-specific. Similarly, we expect that the NPs of the cell-free supernatant obtained from EPN symbiont bacteria will affect them during their feeding activity, causing death. The objectives of this study were (i) to investigate the effect of the foam produced by *P. spumarius* on EPN activity and to evaluate (ii) the EPN virulence and (iii) the symbiont bacterial cell-free supernatant’s toxicity against *P. spumarius* nymphs.

## 2. Materials and Methods

### 2.1. Collecting and Rearing of Organisms

Since an ongoing patent (CSIC) protects the rearing of *P. spumarius* [38], we performed all the experiments using nymphs of *P. spumarius* collected in the field. In periodic samplings during April-May 2019–2020, we collected plants with signs of foam, mostly *Carduus acanthoides* (Asterales: Asteraceae), in weeds adjacent to vineyards located in ‘La Grajera’ (Logroño, La Rioja, Spain, 42°26′ N and 2°30′ W) and belonging to the Government of La Rioja. In the laboratory, we kept the plant material at room temperature and under natural light conditions until the collection of the nymphs of *P. spumarius* for the experiments on the same day as the capture.

We evaluated four EPN species against nymphs of *P. spumarius*: *Steinernema feltiae, S. carpocapsae, S. riojaense*, and *Heterorhabditis bacteriophora* (Table 1). EPNs were cultured in last-instar larvae of *Galleria mellonella* (Lepidoptera: Pyralidae) reared at the Instituto de Ciencias de la Vid y del Vino (Logroño, Spain). The IJs were recovered in tap water upon emergence, stored at 12–14 °C, and used within two weeks after harvest [39]. We completed the molecular identification of all EPN populations following the procedures described by Blanco-Pérez et al. [40]. Briefly, we mechanically disaggregated ~500 IJs employing sterile blue pestles assembled in a pellet mixer (VWR International, Lutterworth, UK). Then, we extracted the DNA with the Speedtools tissue DNA extraction kit (Biotools, Madrid, Spain), analyzed it for quality and quantity using a Nanodrop system (Thermo Scientific 2000C spectrophotometer, provided by Actylab, Logroño, Spain), and stored it at −20 °C until use. For each EPN species, the ITS rDNA region was amplified using universal primers and following the protocols described by Campos-Herrera et al. [41]. All runs contained a negative control by adding mQ water (Milli-Q Water System, Millipore S.A., Molsheim, France) instead of DNA template. Hereafter, the PCR was verified through electrophoresis in 2% agarose gel in TBE (pH 8.0 ± 0.1) to ensure the expected PCR size. Later, individual bands were cut and cleaned (SpeedTools Tissue DNA Extraction kit, Biotools, Madrid, Spain), sequenced, aligned with the software Geneious (R.6.1.5., Biomatters, Inc., Auckland, New Zealand), compared to reported sequences using Blast (http://blast.ncbi.nlm.nih.gov), and submitted to Genbank (Table 1).

The symbiotic bacteria associated with the tested EPN species comprised three *Xenorhabdus* species (*X. bovienii*, *X. nematophila*, and *X. kozodoii*) and one *Photorhabdus* species (*P. laumondii* subsp. *laumondii*) (Table 1). To isolate them, we exposed~500 IJs of each EPN species (inoculated in 100 μL of distilled water) to 5% NaClO for 2–5 min. Later, after thoroughly washing with distilled water, we mechanically disaggregated them in a 50:50 (*v*/*v*) suspension of distilled water and nutritive broth (VWR, Chemicals, Barcelona, Spain), employing sterile blue pestles assembled in a pellet mixer. Then, we seeded 50 μL of each nematode–bacterium complex suspension on three Petri dishes with Nutrient Agar (NA), Bromothymol blue (Alfa Aesar, Kandel, Germany), and 2,3,5-Triphenyl tetrazolium chloride (TTC, VWR, Chemicals, Barcelona, Spain) (NBTA plates) [42], supplemented with Ampicillin (50 mg/mL) (PanReac AppliChem, ITW Reagents, Barcelona, Spain). We stored the Petri dishes for 48 h under controlled conditions (25 ± 2 °C, 20% RH in the dark) before selecting those colonies of morphology associated with most *Xenorhabdus* [43] and *Photorhabdus* [31] species (rounded, smooth margins, and colorant absorption capacity). To obtain pure colonies, we seeded them in NTBA plates. Subsequently, we inoculated single colonies of each pure culture in Triptone Soya Broth (TSB) (VWR Chemicals, Barcelona, Spain), also supplemented with Ampicillin (50 mg/mL), maintaining the liquid cultures for 16 h under stirring (150 rpm) at 22 °C in the dark. We used an aliquot of each suspension to verify the absence of the catalase enzyme of the *Xenorhabdus* strains [27]. Additionally, we checked under a microscope the morphology of bacilli and its mobility using the flagellum. A second aliquot of each bacterial suspension was concentrated and saved for DNA extraction, performed with the Speedtools tissue DNA extraction kit (as described above), and the rest stored at −80 °C in aliquots of 300–400 µl in 30–35% glycerol. We used universal primers to amplify the 16S rDNA region [44].

### 2.2. Production of Cell-Free Supernatant from the Symbiotic Bacteria of Entomopathogenic Nematodes

The bacterial stock was initiated from a single colony of each of the four bacteria, inoculated in liquid media, and grown for 16 h at 25 °C ± 2 °C in darkness under agitation at 150 rpm. Aliquots of 500 µL were stored at −80 °C for each bacterium. Then, we inoculated 100 µL of the aliquots to produce cell-free supernatant in 250 mL of TSB (two 500 mL Erlenmeyer per bacteria). A volume of 50 µL was also seeded in NBTA plates to verify the growth of pure bacteria. We incubated the Erlenmeyer on a shaker for three days under aerobic and dark conditions, at 150 rpm and 25 ± 2 °C, in darkness. Subsequently, we centrifuged the bacterial media at 25830 g and 4 °C for 40 min, and the supernatant was filtered through a 0.22 µm sterile pore filter. An aliquot of this filtrate was cultured on NBTA plates to verify the absence of bacteria. The filtrate was defined as cell-free supernatant and subsequently used in toxicity tests. The TSB media used as controls were also filtrated to follow the same protocols as treatments. The material was used immediately upon filtration.

### 2.3. Evaluation of Entomopathogenic Nematode Virulence after Exposure to Foam Produced by Philaenus spumarius

We evaluated the IJ virulence when exposed to the foam produced by the nymphs of *P. spumarius* for 24 and 72 h for *S. feltiae* and only for 24 h for *H. bacteriophora*. We employed two 24-multi-well trays (Corning, NY, USA) per treatment, using 12 interleaved wells per tray. In each well, we added 0.5 g of sterilized sand (pure sand, Vale do Lobo, Loulé, Portugal), 1 cm^2^ of a leaf of *C. acanthoides* (Finca de La Grajera, La Rioja, Spain), and the volume of foam corresponding to (approximately) the production of a single nymph of *P. spumarius*. Immediately after, 20 µL of water with 3 IJs was inoculated inside the foam. The control treatments followed the same procedure but without the presence of the foam. In addition, we included two treatments without nematode application, one with only water and another with foam only, as controls in the subsequent study of infectivity against *G. mellonella*. After incubation under controlled conditions (80% RH, 20 °C/16 h light, and 14 °C/8 h dark, on-ramp/for 24 or 72 h), we added *G. mellonella* larvae to each well. We checked the larval mortality daily for six days. The experiment was conducted twice with freshly produced foam, plant material, larvae of *G. mellonella*, and nematode cultures.

### 2.4. Evaluation of Entomopathogenic Nematode Virulence and Bacterial Cell-Free Supernatant Toxicity against Philaenus spumarius

We placed five nymphs of *P. spumarius* (using hairbrush 000 sizes) in 55 mm Petri dishes (*n* = 10) with two filter papers (Whatman no.1) arranged on the inner faces. The final volume applied per filter paper was 500 μL. First, to favor nymph settlement, we moistened them with distilled water, 400 μL for the EPN virulence test and 450 or 425 μL (depending on the selected supernatant dilution, see below) for the cell-free supernatant toxicity test. Then, in the EPN test, we inoculated a total of 75 IJs per Petri dish, applied half on the top and half on the bottom filter paper in 100 μL suspension. In the case of the cell-free supernatant toxicity test, we applied 50 or 75 µL of the supernatant (to obtain a concentration of 1:10/1: 6.67 metabolite concentration) to each of the filter papers. In all the cases, we included control treatments containing only water or equal proportions of sterile and filtered culture media. We also included for the cell-free supernatant experiment the mixed treatments *X. bovienii + X. nematophila* (1:1) and *X. nematophila + P. laumondii* subsp. *laumondii* (1:1) to study the interaction of their metabolites. All the plates, closed with parafilm, were incubated under controlled conditions with an increase in temperature to simulate regional spring temperatures (±60% RH, 20 °C/10 h light and 14 °C/14 h darkness) (https://www.larioja.org/agricultura/es/informacion-agroclimatica/red-estaciones-agroclimaticas-siar) (accessed on 29 March 2021). We applied 50 µL of a sucrose solution (1 g in 10 mL distilled water) per Petri dish every two days to allow their feeding. We checked the nymphal mortality daily for six days. The experiment was conducted twice with freshly produced bacterial cell-free supernatant, nematodes, and insects.

### 2.5. Statistical Analyses

We ran general linear models (GLM), with a binomial distribution (logit-link function), for the pair-treatment comparisons (control versus treatment) testing the impact of the foam produced by P. *spumarius* on EPN virulence against G. mellonella last-instar larvae as well as the IJ virulence and bacterial cell-free supernatant toxicity on nymphs of P. *spumarius*. To evaluate the nature of the combination of NPs (antagonistic, no-interaction/additive, or synergistic), we followed the formulae proposed by Shapiro-Ilan et al. [45] and Ansari et al. [46]. We compared the expected and observed nymph mortalities for each single NP and mixed NP. The expected mortalities (M_E_) were calculated as M_E_ = M_T1_ + [M_T2_ × (1 − M_T1_)] when different NPs were combined. We ran an χ^2^ test for the expected and observed mortalities [i.e., χ^2^ = (M_T1T2_ − M_E_)^2^/M_E_, where M_T1T2_ is the observed mortality for each single NP]. These values were matched with the χ^2^ table for one degree of freedom (*p* = 0.05) so that χ^2^ < 3.8415 indicated additive interaction and χ^2^ > 3.8415 non-additive (antagonist or synergist) interaction. Thus, the interaction was considered synergistic if M_T1T2_ − M_E_ > 0, and antagonistic if M_T1T2_ − M_E_ < 0 [45,46]. We performed all analyses with SPSS 25.0 (SPSS Statistics, SPSS Inc., Chicago, IL, USA), using *p* < 0.05 for assessing statistical differences. We used least-square means ± SE as descriptive statistics.

## 3. Results

**Figure 1:**
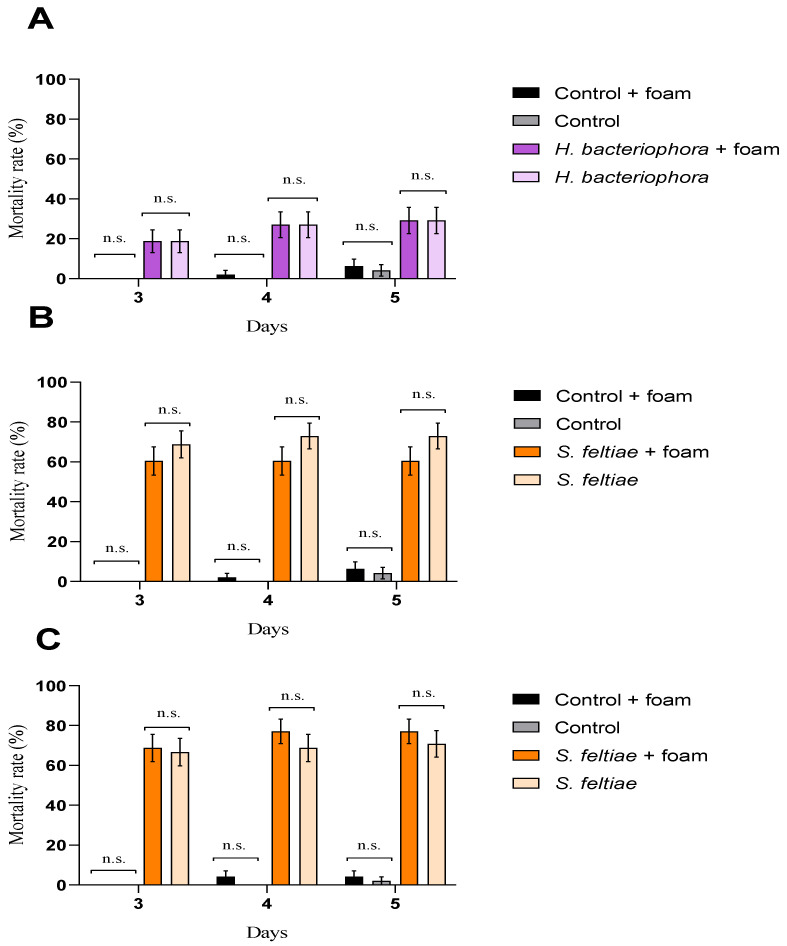
Entomopathogenic nematode pathogenicity against *Galleria mellonella* larvae after exposure to the foam produced for *Philaenus spumarius*. Cumulative larval mortality (three to five days) at (**A**) 24 h exposure for *Heterorhabditis bacteriophora*, (**B**) 24 h exposure for *Steinernema feltiae,* and (**C**) 72 h exposure for *S. feltiae.* No significant differences (n.s.) (*p* < 0.05) in general linear model testing within pair-treatment comparisons of exposure and no exposure to foam were found. Values are least-square means ± SE.

The foam produced for nymphs of *P. spumarius* affected neither EPN pathogenicity nor *G. mellonella* larvae, independently of the EPN species evaluated or the time of exposure (Figure 1).

**Figure 2:**
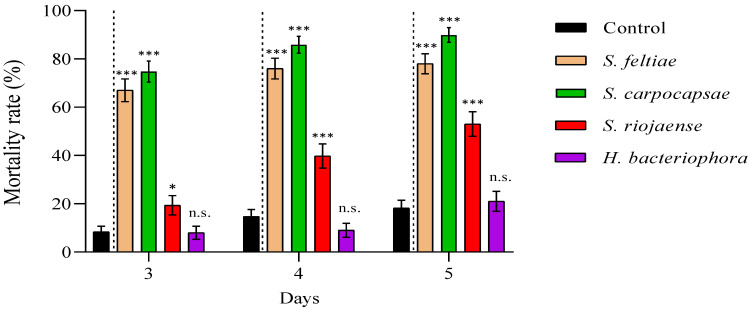
Entomopathogenic nematode (EPN) pathogenicity against *Philaenus spumarius* nymphs. Cumulative larval mortality (three to five days) for the EPN species *Steinernema feltiae*, *S. carpocapsae*, *S. riojaense, Heterorhabditis bacteriophora*, and the absence of nematodes (control). Asterisks indicate significant differences at *** *p* < 0.001, * *p* < 0.05, and n.s., not significant, for generalized linear models testing within pair-treatment comparisons of inoculations and no inoculations (control) of EPNs. Values are least-square means ± SE.

**Figure 3:**
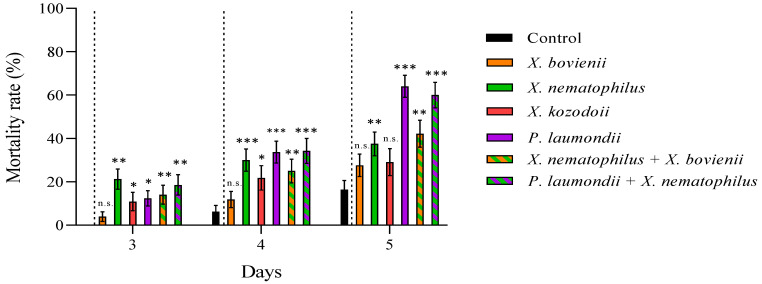
Cell-free supernatant 1:10 diluted against *Philaenus spumarius* nymphs. Cumulative larval mortality (three to five days) for the symbiont bacterial species *Xenorhabdus bovienii*, *X. nematophilus X. kozodoii*, *Photorhabdus laumondii*, and the combinations of *X. nematophilus + X. bovienii* and *P. laumondii* + *X. nematophilus*. Asterisks indicate significant differences at *** *p* < 0.001, ** *p* < 0.01, * *p* < 0.05, and n.s., not significant, for generalized linear models testing within pair-treatment comparisons of inoculation and no inoculation (control) of cell-free supernatants. Values are least-square means ± SE.

**Figure 4:**
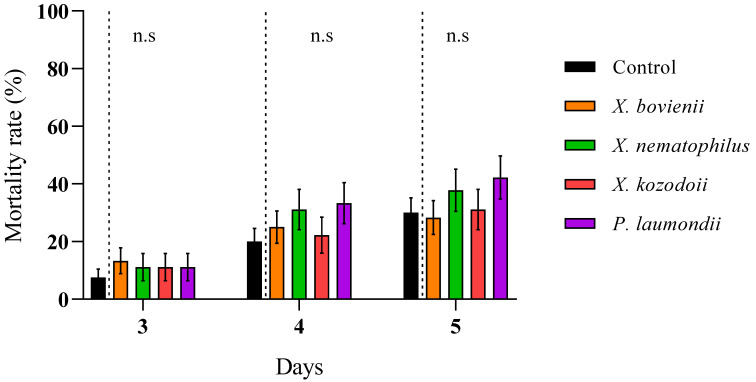
Cell-free supernatant 1:6.67 diluted against *Philaenus spumarius* nymphs. Cumulative larval mortality (three to five days) for the symbiont bacterial species *Xenorhabdus bovienii*, *X. nematophilus X. kozodoii*, *Photorhabdus laumondii*, and the combinations of *X. nematophilus + X. bovienii* and *P. laumondii* + *X. nematophilus*. No significant differences (n.s.) (*p* < 0.05) for general linear model testing within pair-treatment comparisons of inoculation and no inoculation (control) of cell-free supernatants were found. Values are least-square means ± SE.

We reported high nymphal mortality rates for the three steinernematid species for all revised days, particularly for *S. feltiae* and *S. carpocapsae* IJs, while we did not observe differences in the mortalities due to *H. bacteriophora* and control (absence of IJs) treatments (Figure 2; Supplementary Material, Table S1). On the other hand, the nymph mortality was strongly dependent on the initial concentration of cell-free supernatant applied: the application of 1:10 dilutions, except for a few cases, significantly increased nymph mortality rates compared to control treatments for all counting days (Figure 3), while 1:6.67 dilutions did not (Figure 4; Supplementary Material, Table S1). Contrary to our observations for IJ inoculations, we reported the highest mortality rates for the application of 1:10 dilution cell-free supernatant from *P. laumondii* subsp. *laumondii,* the symbiont bacteria isolated from *H. bacteriophora.* (Figure 3). For the natural products derived from *Xenorhabdus* spp., we observed differences only for the cell-free supernatant from *X. nematophilus* (isolated from *S. carpocapsae*) for all counting days and from *X. kozodoii* (isolated from *S. riojaense*) at day three and four after application (Figure 3; Supplementary Material, Table S1). Both cell-free supernatant combinations resulted in additive effects (Table 2).

## 4. Discussion

This study shows the potential of EPNs and the application of their symbiont bacterial cell-free supernatant to control nymphs of *P. spumarius.* First, we observed that the foam produced by *P. spumarius* nymphs did not affect EPN virulence after 24 and 72 h of exposure, despite previous records on the nature and function of this foam. Other studies proposed that cercopid foam creates a microhabitat that protects against desiccation, extreme temperatures, and predatory and parasitic enemies [15]. Indeed, the only parasite of cercopid nymphs reported is a nematode in the family Mermithidae [47]. In this line, laboratory bioassays showed that some natural spittlebug foams, and a synthetic mixture composed of representative compounds identified in it, are repellent to ants and produce topical irritation in cockroaches [16]. However, our results showed that the foam was not deleterious to IJs. Since the foam could also protect the applied IJs and facilitate their movement to locate the nymphs, our results suggest that the direct application of EPN suspensions in the spit–nymph complex might be compatible and hence a promising method to control the pest in crops.

It is noteworthy that the opposite results were obtained for the insecticidal effect against *P. spumarius* nymphs for IJs and cell-free supernatants of the same EPN species. Thus, after five days of exposure, we reported over 80% nymphal mortality rates for *S. carpocapsae* and *S. feltiae* IJ applications, while they did not reach 50% for the NP applications of their symbiont bacteria. Conversely, the pathogenicity observed for *H. bacteriophora* IJs was not significantly higher than that obtained for the controls, while their NP applications surpassed 60% mortality at five days of exposure. These results illustrate the differences between EPN species in their efficiency when locating and penetrating susceptible hosts, and how the environment might modulate their virulence. For example, temperature is a factor that affects EPN infectivity and reproduction [48]. The virulence studies against *P. spumarious* nymphs were performed in a temperature range of 14–20 °C to simulate typical spring changes in La Rioja (Spain). These changes in the temperature could favor the activity of certain species. For example, *S. feltiae* infection can be achieved from 8 to 30 °C and reproduction from 10 to 25 °C, while the *H. bacteriophora* range included higher values, from 10 to 32 °C and 15 to 30 °C, respectively [48]. The limited infectivity observed for *H. bacteriophora* IJs could be due to the stress when the temperature decreased to 14 °C. Surprisingly, *S. carpocapsae* IJs exhibited high mortality rates against *P. spumarius* nymphs even if the temperature range for successful infectivity and reproduction was similar to that reported for *H. bacteriophora.* It is plausible that the differences in virulence observed in this study are due to the broader/limited range of temperature for infectivity and reproduction that could characterize the selected population. Moreover, several EPN species could likely show better compatibility with this host. Regardless of whether the best infectivity is related to the EPN population employed, the fit with the host, or a combination of both, our results showed the compatibility of certain EPN species to fight against the nymphs of *P. spumarius.*

On the other hand, we verified, for the first time, the insecticidal activity of *Xenorhabdus* and *Photorhabdus* cell-free supernatants against *P. spumarius* nymphs when ingested orally. Nymphal mortality was observed the day after the application of the sucrose suspension that allowed them to feed. The wide variety of products released by EPN symbiont bacteria perform different functions for the nematode–bacteria complex. The toxicity of cell-free supernatants extracted from EPN symbiont bacteria against an ample range of insects is well known [49]. Indeed, *Xenorhabdus* [50] and *Photorhabdus* [51,52] display different gene clusters related to their bioactivity that, when combined, establish a suitable niche to survive and reproduce within the host cadaver. This diversity of natural compounds makes them a powerful tool for exploring new bioproduct development to be used as biopesticides. However, additional studies are required to establish the specific compounds responsible for the insecticidal effect on selective targets and, in particular, *P. spumarius*.

To improve the insecticidal effect of individual bacterial cell-free applications, we combined and tested the cell-free supernatant proceeding from different symbiont bacterial species. We observed that none of the two mixed treatments, *X. bovienii* + *X. nematophila* and *X. bovienii* + *P. laumondii* cell-free supernatants (1:1), enhanced the insecticidal impact over the prevailing metabolite, showing a final additive effect. Further investigation to enhance this activity might warrant attention. For example, the natural product generation might differ if two or more bacteria species are combined at the beginning of the fermentation. Moreover, different proportions to the 50:50 investigated herein can increase nymph mortality. In this line, our results revealed the importance of fine-tuning for bacterial cell-free supernatant applications. Thus, we observed that 1:10 dilution applications were bioactive against *P. spumarius* nymphs, while, at a slightly higher concentration (1:6.67), the possible insecticidal effect was masked by the TSB oral toxicity observed in the controls. Hence, additional studies are required to select the best bacterial NPs, concentration rates, and application procedures to optimize the use of this promising biotool.

To the best of our knowledge, no previous study relates the direct application of EPNs and the use of NPs from their symbiotic bacteria to control the same pest. Since *P. spumarius* is the most relevant vector of *X. fastidiosa* in the EU, there is an urgent need to provide tools to reduce its propagation, particularly in organic production, for which the use of pesticides is strictly limited. Furthermore, EPN implementations to fight this vector–disease complex are highly viable as there are numerous commercial products based on them [53]. However, additional studies are required to evaluate the impact of EPN or cell-free supernatant application on *P. spumarius* nymphs infected with *X. fastidiosa*. Advances in this knowledge will contribute to extending the strategies currently proposed by the EU, focused on host removal, vector control, and restrictions on the production and transport of plant materials, for the eradication or containment of this disease.

## 5. Conclusions

Our results showed that the foam produced by nymphs of the spittlebug *P. spumarius* did not affect EPN virulence. Indeed, *steinernematid* IJs caused significant nymphal mortality rates while *H. bacteriophora* not. Moreover, the cell-free supernatant obtained from their symbiont bacteria showed toxicity against *P. spumarius* nymphs, particularly for *Photorhabdus* species. The knowledge gained herein has opened a new avenue for advances in innovative approaches to complement traditional strategies. These natural products are promising biopesticides that require a deep understanding due to their broad potential for controlling arthropod pests in sustainable agriculture. Therefore, further research is needed to isolate, identify, and characterize the metabolites produced by the EPN symbiotic bacteria, but also to prove that their application will be safe for non-target organisms, plants, and the environment before being used as biopesticides.

## Figures and Tables

**Table 1:** Entomopathogenic nematode (EPN) and symbiotic bacterial species tested against nymphs of *Philaenus spumarius*.

EPN Species	Population	ITS-GenBank Accession	Bacterial Species	ITS-GenBank Accession
*Steinernema feltiae*	RM-107	MW480131	*Xenorhabdus bovienii*	MW467374
*Steinernema carpocapsae*	All	MW574913	*Xenorhabdus nematophilus*	MW574906
*Steinernema riojaense*	RM-30	MK503133	*Xenorhabdus kozodoii*	MW467375
*Heterorhabditis bacteriophora*	RM-102	MW480132	*Photorabhdus laumondii* subsp. *laumondii*	MW574908

**Table 2:** Interactions of the mixed cell-free supernatant applications *Xenorhabdus bovienii* + *X. nematophilus* and *X. nematophilus* + *Photorhabdus laumondii*. Expected mortality (ME) calculated as ME = MT1 + [MT2 x (1 − MT1)], where MT1 and MT2 are the observed mortality rates (%) recorded for single cell-free supernatant applications. Interactions were based on χ^2^ ratio between expected and observed mortalities (χ2 = (MT1T2 − ME)2/ME, where MT1T2 is the observed mortality for each single application).

Combinations	Observed Mortality (%)	Expected Mortality (%)	χ2	Interaction
*X. nematophilus + X. bovienii*	42	43	3.20	Additive
*P. laumondii + X. nematophilus*	60	68	2.33	Additive

## Supplementary Materials

The following are available online at https://www.mdpi.com/article/10.3390/insects12050448/s1, Table S1. Results from generalized linear mixed models testing within pair-treatment comparisons (treatment vs. controls) for the impact of entomopahtogenic nematodes (EPNs) and cell-free supernatants (SM) of their symbiont bacteria (applied at two concentrations) on *P. spumarius* nymphs. Asterisks indicate significant differences at *** *p* < 0.001, ***p* < 0.01, * *p* < 0.05, and n.s., not significant.
