# Supplementary material for: Insecticidal Effect of Entomopathogenic Nematodes and the Cell-Free Supernatant from Their Symbiotic Bacteria against Philaenus spumarius (Hemiptera: Aphrophoridae) Nymphs
Source: Insects. 2021 May 14;12(5):448. doi: 10.3390/insects12050448 (PMC8156950; doi:10.3390/insects12050448)
Supplement: Supplementary file 1 [file insects-12-00448-s001.zip › insects-1179440-supplementary.pdf]

Table S1. Results from generalized linear mixed models testing within pair-treatment comparisons (treatment vs. controls) for the impact of entomopahtogenic nematodes (EPNs) and cell-free supernatants (SM) of their symbiont bacteria (applied at two concentrations) on *P. spumarius* nymphs. Asterisks indicate significant differences at ***$P < 0.001$, **$P < 0.01$, *$P < 0.05$, and n.s., not significant.

		Day 3		Day 4		Day 5	
	Pair-treatment comparison (treatment vs. control)	χ^2	P	χ^2	P	χ^2	P
EPNs	*S. feltiae*	70,491	***	76,652	***	72,865	***
	S. carpocapsae	83,603	***	91,33	***	86,051	***
	S. riojaense	5,963	*	18,398	***	30,077	***
	H. bacteriophora	0,012	n.s.	1,726	n.s.	0,299	n.s.
SM 1 : 10	*X. bovienii*	1,063	n.s.	1,39	n.s.	2,78	n.s.
	X. nematophilus	11,988	**	12,499	***	8,53	**
	X. kozodoii	5,092	*	6,284	n.s.	2,99	n.s.
	P. laumondii	6,581	*	15,442	***	34,42	***
	X. nematophilus + X. bovienii	7,233	**	8,581	**	10,99	***
	X. nematophilus + P. laumondii	10,128	**	15,088	***	27,19	***
SM 1 : 6.67	*X. bovienii*	1,265	n.s.	0,496	n.s.	0,46	n.s.
	X. nematophilus	0,462	n.s.	1,923	n.s.	0,787	n.s.
	X. kozodoii	0,459	n.s.	0,086	n.s.	0,017	n.s.
	P. laumondii	0,459	n.s.	2,685	n.s.	1,886	n.s.
